# Moderation of marital status and living arrangements in the relationship between social participation and life satisfaction among older Indian adults

**DOI:** 10.1038/s41598-022-25202-5

**Published:** 2022-11-29

**Authors:** Varsha P. Nagargoje, K. S. James, T. Muhammad

**Affiliations:** International Institute for Population Sciences (IIPS), Mumbai, 400088 India

**Keywords:** Geriatrics, Health policy, Public health, Quality of life

## Abstract

Social participation is considered one of the central components of successful and healthy aging. This study aimed to examine the moderating role of marital status and living arrangement with social participation and its association with life satisfaction of older Indian adults. Samples of 31,464 individuals aged ≥ 60 years were extracted from the Longitudinal Ageing Study in India, wave-1. Descriptive statistics, bivariate analysis, and multivariable linear regression were performed for the analysis. The moderation effect of marital status and living arrangements on the relationship between social participation and level of life satisfaction among Indian older adults were also analyzed. Overall, life satisfaction among older men was relatively higher than older women in this study. Older adults’ involvement in social participation [β = 0.39, *p* < 0.05], being in marital union [β = 0.68, *p* < 0.001] and co-residing either with spouse [β = 1.73, *p* < 0.001] or with other family members [β = 2.18, *p* < 0.001] were positively related to their greater life satisfaction. Interaction of social participation with marital status showed that participating in social activities can boost life satisfaction only among married older people. Further, moderation effect of social participation with living arrangements showed that older adults who were not involved in social participation but living with a spouse or any other household members had higher life satisfaction, and again participation in social activities increased their life satisfaction to a greater level. The establishment of social clubs and advocating social policies oriented toward meaningful social connections are highly needed, especially for older Indians living alone or currently not in a marital union, which will help to enhance their overall life satisfaction.

## Introduction

The population in India is undergoing visible transitions and is currently aging at an accelerated rate. According to the Census 2011, around nine percent of the total population in India (i.e., 104 million) aged 60 years and over^1^. Projections of the United Nations pointed out that this proportion will reach around 19 percent (i.e., 330 million) by 2050, threefold the number identified by the Census of India, 2011^2^. Although people are surviving for longer ages, it is important to have a healthy and active life expectancy. The previous literature documented that life satisfaction of older adults in Asia is by virtue of several determinants like social functioning, health status, housing satisfaction, economic status, social support, cognitive ability, marital status, living arrangement, religiosity, education level, standard of living, family institution and good governance, etc.^3,4^. Some literature also confirms that social participation promotes successful and healthy aging^5–8^ and benefits older individuals’ quality of life and their life satisfaction^9,10^.

Using Walker and Avant’s 8-step method of concept analysis, Aroogh and Shahboulaghi^11^ have recently provided a more comprehensive definition of social participation. According to their analysis, older individuals’ social participation consists of community-based activities and interpersonal interactions based on resource sharing, active participation, and individual satisfaction. Moreover, older adults’ social participation within communities and within family groups is linked to a sense of belonging and interpersonal social connections^12^, which results in aging successfully in later life^6,7,13^. However, life-cycle transitions like retirement, loss of spouse/partner, solo living and separation from friends or family, loss of mobility, declining physical and mental health conditions can change the social participation pattern of older people. Further, as age progresses, their social networks may shrink^14^ due to the death of friends and/or their partners. Such reduced social contact can put older adults at increased risk of loneliness and social isolation, which in turn affect the satisfaction and quality of aged people’s lives.

The activity theory of aging by Havighurst and Albrecht (1953) proposes that successful aging occurs when older adults remain active and maintain social interactions. This theory was considered the basis for the current study as many researchers in the field of gerontology favored it. They believed that older people should maintain their middle-age activities as long as possible and then have to find substitutes for those activities they must give up. For instance, substitute for work after retirement; substitute for friends and loved ones whom they lose by death; substitute for clubs and associations which they must give up. These substituted activities help older people to replace their lost life roles. Activity theory assumed that involvement in social activities improves life satisfaction in old age^15,16^ and several empirical studies support the hypothesis of activity theory of aging^17–22^.

Including Gilmour (2012), researchers suggested that along with frequency of social participation, the quality of social network also matters for improved well-being of older adults^18,23–25^. A study based on the Swiss older population validate the result that having rich and varied social participation can improve the individuals’ level of life satisfaction^19^. Although people with greater social participation have fewer chances of feeling loneliness and dissatisfaction, various health conditions of aged people, such as self-rated health (SRH)^26^, quality of life^18,27^, functional ability^28^, and multimorbidity/chronic illness^29,30^, can influence older adults’ opportunities for social participation. Findings from few studies suggested that older people living with a chronic illness or functional disability are at increased risk of restricted social participation and increased loneliness^31,32^ which ultimately affect the quality of life.

Furthermore, living arrangements act as a powerful function in determining level of life satisfaction of older adults. Kim et al.^24^ found that among physically disabled Korean older adults, solo living older persons and those living with others have poorer life satisfaction than those living with a spouse. Another study among Malaysian population is in agreement that co-residency with children, and living specifically with a spouse, was associated with better life satisfaction compared to living alone^33^. Li and colleagues^22^ studied Chinese older adults’ level of life satisfaction and concluded that increased social participation potentially enhances Chinese older adults’ life satisfaction, but their marital status did not posit any impact on the level of life satisfaction.

**Figure 1:**
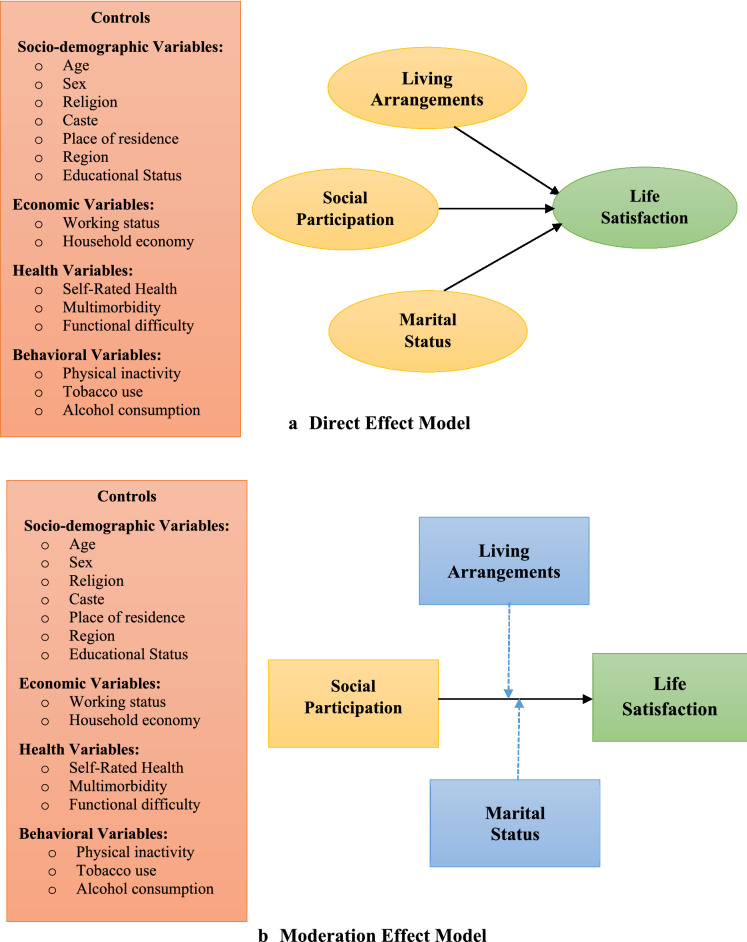
Model (**a**) Direct effect of social participation, marital status and types of living arrangements on Indian older adults’ life satisfaction Model (**b**) Moderation effect of social participation with marital status and types of living arrangements on Indian older adults’ life satisfaction.

Some researchers have made an attempt to examine the role of social networks on life satisfaction among older adults in India^34–36^. However, these studies targeted a small subset of a group and could not represent India’s entire older population. Moreover, to authors’ knowledge, no attention has been paid to how older adults’ marital status and living arrangements moderate the relationship between older adults’ social participation and life satisfaction which could display the overall picture of the well-being of the aging population in India. Therefore, the current study aimed to explore the relationship between social participation, marital status, living arrangements and life satisfaction among older Indian adults. Based on the reviewed literature and theoretical background mentioned above, and the conceptual framework summarized in Fig. 1, the following research hypotheses are proposed (i) engagement in social participation is related to greater life satisfaction among Indian older adults, and (ii) marital status moderates the relationship between social participation and Indian older adults’ life satisfaction (iii) living arrangements of older Indian adults moderate the association between social participation and their life satisfaction.

## Methods

### Study design and sample

A cross-sectional study design was adopted in this study. Data for the study were drawn from the Longitudinal Ageing Study in India (LASI) wave 1 that was collected during 2017–18. LASI is a nationally representative survey of over 72,000 individuals aged 45 and above across all states and union territories (UTs) of India. The main objective of the survey was to study the health status and the socioeconomic well-being of older adults in India. The present study is conducted among older adults aged 60 years and above with a total sample size of 31,464 older adults (men-15,098 and women-16,366).

### Procedure

For the selection of samples, LASI survey followed a multistage stratified area probability cluster sampling design where in rural areas a three-stage and in urban areas a four-stage sampling design were adopted. In each state/UT, the first stage involved the selection of Primary Sampling Units (PSUs), that is, sub-districts (Tehsils/Talukas), and the second stage involved the selection of villages in rural areas and wards in urban areas in the selected PSUs. In rural areas, households were chosen from selected villages in the third stage. However, sampling in urban areas involved an additional stage. Specifically, in the third stage, one Census Enumeration Block (CEB) was randomly selected in each urban area and then in the fourth stage, households were selected from this CEB. The goal was to select a representative sample in each stage of sample selection. Further, an individual survey schedule was administered to each consenting respondent aged 45 and above and their spouses (irrespective of age) in the sampled households. In addition, the LASI includes an individual module on biomarkers and direct health examination. The detailed methodology, with the complete information on the survey design and data collection, was published in the survey report^37^. The data were anonymized before its use and all the methods were performed in accordance with relevant guidelines and regulations. The survey agencies that conducted the field survey for the data collection have collected prior consent from the respondents. The Indian Council of Medical Research (ICMR) extended the necessary guidelines and ethics approval for undertaking the LASI survey.

### Measures

#### Outcome variable

The Satisfaction with Life Scale (SWLS) is a 5-item instrument designed to measure global cognitive judgments of satisfaction with one’s life^38^. Life satisfaction among older adults was assessed using the questions a. In most ways my life is close to ideal; b. The conditions of my life are excellent; c. I am satisfied with my life d. So far, I have got the important things I want in life; e. If I could live my life again, I would change almost nothing. The responses were 7-point Likert scale, ranging from 1 to 7 as strongly disagree, somewhat disagree, slightly disagree, neither agree nor disagree, slightly agree, somewhat agree and strongly agree. Using the responses to the five statements regarding life satisfaction, a continuous scale was constructed with a score ranging from 5 to 35. Past research has shown that the scale’s internal consistency was high (α = 0.87) and two-week test–retest reliability was r = 0.85. The Cronbach’s alpha in the current study was 0.89. A score of 20 and above was considered the cut-off for high life satisfaction and the continuous scale was used in the multivariable analysis.

#### Main explanatory variables

##### Social participation

Following the previous studies^39,40^, survey questions based on participation in social activities were assessed to generate this variable. The activities included eating out of the house, going to park/beach, visiting relatives/friends, attend cultural performances/shows/cinema, attending religious functions/events, and attending community/political/organization group meetings, were included (Cronbach’s alpha: 0.6). If the respondents reported participating in any of the above activities at least once in a month, they were considered as having social participation, and the variable was recoded as 1 'yes' (1 = at least once in a month), and 0 'no' (0 = rarely or never).

##### Marital status

It was coded as 'currently married' and 'currently unmarried'. Currently unmarried included widowed/ divorced/ separated/ never married^41^.

##### Living arrangements

Types of living arrangements were categorized into three parts: 'living alone', 'living with a spouse', and 'living with others'. This category consists of various combinations of older adults’ co-residential living i.e., living with spouse and children, living with spouse and other relatives, and living with children and/or other relatives^42^.

#### Socio-demographic characteristics

Following the above mentioned literature, several socio-demographic variables were selected and included in the current analysis. Age was categorized into age groups of '60–69 years', '70–79 years', and '80+ years'. Sex was coded as 'male' and 'female'. Educational status was coded as 'no education/primary', 'secondary', and 'higher'. Working status was coded as 'never worked', 'currently not working', 'working', and 'retired'.

#### Health and behavioral characteristics

Multiple health and behavioural variables were considered in the study since they are shown to be potential confounders. Physical activity status was categorized as yes (every day, more than once a week, once a week, one to three times in a month) and no (hardly ever or never). The question through which physical activity was assessed was "How often do you take part in sports or vigorous activities, such as running or jogging, swimming, going to a health centre or gym, cycling, or digging with a spade or shovel, heavy lifting, chopping, farm work, fast bicycling, cycling with loads?"^37^.

Self-rated health (SRH) was coded as 'good' which includes excellent, very good and good whereas 'poor' includes fair and poor^43^.

Multimorbidity is referred to as the coexistence of two or more chronic physical health conditions in the same individual^44^. For this analysis, nine chronic health conditions were included, namely hypertension, chronic heart diseases, stroke, any chronic lung disease, diabetes, cancer or malignant tumour, any bone/joint disease, neurological/psychiatric disease, or high cholesterol^37^. The diseases were self-reported as was assessed through the question "Has any health professional ever diagnosed you with the following chronic conditions or diseases?". Tobacco use and alcohol consumption were coded as 'no' and 'yes' referring to ever use of tobacco or alcohol.

#### Household characteristics

The following household and community-related variables were included as control variables in this study. The monthly per-capita consumption expenditure (MPCE) quintile was assessed using household consumption data. Sets of 11 and 29 questions on the expenditures on food and non-food items, respectively, were used to canvas the sample households. Food expenditure was collected based on a reference period of seven days, and non-food expenditure was collected based on reference periods of 30 days and 365 days. Food and non-food expenditures have been standardized to the 30-day reference period. The MPCE is computed and used as the summary measure of consumption^37^. The variable was divided into five quintiles, i.e., from 'poorest' to 'richest'. Religion was recoded as 'Hindu', 'Muslim', 'Christian', and 'Others'. Caste was recoded as 'Scheduled Tribe', 'Scheduled Caste', 'Other Backward Class', and 'others'. The place of residence was coded as 'urban' and 'rural'. The geographical regions of the country were coded as 'North', 'Central', East', 'Northeast', 'West', and 'South'.

### Statistical analyses

In this study, descriptive statistics and bivariate analysis has been performed to assess the prevalence of life satisfaction along with all key explanatory variables, marital status, living arrangements and social participation. Further, multivariable linear regression analysis was used to test the research hypotheses of the study. The results are presented in the form of beta coefficient with a 95 percent confidence interval (CI). Also, individual weights were used to make the estimates nationally representative. For all the analyses, STATA version 14 has been used^45^.

The multivariable regression analysis provides three models to explain the adjusted estimates using LASI, wave 1 sample of individuals 60 years and above. Model-1 demonstrates the effect of three key variables, i.e., social participation, marital status, and living arrangements, on life satisfaction while adjusted for the selected control variables. Model-2 analysed the moderating effect of marital status with regard to social participation and life satisfaction of older adults. Lastly, model-3 provides the moderating effects of living arrangements in the link between social participation and life satisfaction among older Indian adults^46^.

### Ethical approval and consent to participate

The survey agencies that conducted the field survey for the data collection have collected prior informed consent (written and verbal) from all the participants. The Indian Council of Medical Research (ICMR) extended the necessary guidance and ethical approval for conducting the LASI survey.

All methods were carried out in accordance with relevant guidelines and regulations by the Indian Council of Medical Research (ICMR).

## Results

### Socioeconomic profile of the older adults

Table 1 represents the descriptive statistics of the sample characteristics. The proportion of older adults in the age group of 80 years and above was 11.29 percent in this study. Men constitute 47.5 percent of the total interviewed respondents. Of all sampled respondents, around three-fourth had either no education or up to primary education, 30 percent reported that they were still engaged in the workforce; about 62 percent were unmarried at the time of the interview. Almost three-fourth of older individuals was co-residing with adult children and other relatives, and around six percent were living alone. More than half of the respondents were engaged in social participation. Of selected respondents, only 31 percent were involved in some physical activity; 76 percent reported good SRH, 24 percent had a multimorbidity, and around 40 percent and 15 percent reported tobacco and alcohol consumption respectively. The proportion of older individuals from the poorest consumption quintile (21.7 percent) was greater than the richest (16.5 percent) category, more than four-fifth of the sample belonged to Hindu religion, and approximately 71 percent were from the rural areas.

**Table 1:** Background characteristics of the study participants, LASI Wave 1, 2017–18.

Background Factors	Total Sample (N = 31,464)	
	(N)	Percent (%)
**Age (in years)**		
60–69	18,974	58.51
70–79	9101	30.20
80+	3589	11.29
**Sex**		
Male	15,098	47.45
Female	16,366	52.55
**Educational status**		
No/primary	22,729	74.02
Secondary	6106	18.24
Higher	2629	7.74
**Working status**		
Never	8784	26.43
Not	10,990	36.45
Yes	8997	29.87
Retired	2693	7.25
**Marital status**		
Currently married	19,920	61.63
Currently unmarried^#^	11,544	38.37
**Living arrangement**		
Living alone	1622	5.68
Living with spouse	6215	20.33
Living with others^##^	23,627	73.99
**Social participation**		
No	14,241	46.83
Yes	17,223	53.17
**Physical activity**		
No	21,653	68.90
Yes	9545	31.10
**SRH**		
Good	23,685	75.79
Poor	7113	24.21
**Multimorbid**		
No	23,576	75.95
Yes	7797	24.05
**Tobacco use**		
No	19,034	59.83
Yes	12,178	40.17
**Alcohol consumption**		
No	25,855	85.41
Yes	5364	14.59
**ADL difficulty**		
No	24,642	76.23
Yes	6694	23.77
**IADL difficulty**		
No	17,449	51.64
Yes	13,846	48.36
**MPCE quintile**		
Poorest	6484	21.70
Poorer	6477	21.71
Middle	6,416	20.95
Richer	6170	19.19
Richest	5917	16.45
**Religion**		
Hindu	23,037	82.20
Muslim	3731	11.30
Others	4696	6.50
**Caste**		
SC/ST	10,313	27.10
OBC	11,886	45.20
Others	9265	27.70
**Place of residence**		
Urban	10,739	29.45
Rural	20,725	70.55
**Region**		
North	5812	12.59
Central	4262	20.95
East	5757	23.64
Northeast	3752	2.97
South	7528	22.68
West	4303	17.17

% Weighted percentage, Individual sampling weights given in LASI wave 1, 2017–18 were applied, counts (N) are un-weighted.

*SRH* self-rated health, *ADL* activities of daily living, *IADL* instrumental activities of daily living, *MPCE* monthly per capita consumption expenditure.

^#^Currently unmarried category comprises widowed, divorced, separated, deserted and never married older individuals.

^##^Category of living with others consists of those older adults who were co-residing either with their spouse and children, living with spouse and other relatives, and living with children and/or other relatives.

### Prevalence of higher life satisfaction among older adults

Table 2 illustrates the bivariate association between older adults’ life satisfaction with different background variables stratified by respondents’ sex. Overall, the proportion of life satisfaction among older men was relatively higher than older women in this study. The life satisfaction increased with greater education levels among older men and women. Retired older persons reported higher life satisfaction compared to other categories of work status. Older individuals who were currently married, residing either with a spouse or other family members, and participating in social activities experienced higher life satisfaction than their counterparts. Older adults engaged in physical activities reported less satisfaction with life. Higher life satisfaction was experienced by older adults who reported good self-rated health, did not have any difficulty performing ADL or IADL, who belonged to the richer and richest MPCE quintile and those lived in urban areas. Overall, around 70 percent men and 66 percent women reported a higher satisfaction with life.

**Table 2:** Bivariate estimates for life satisfaction by background characteristics among older adults, LASI Wave 1, 2017–18.

Variables	Men		Women	
	Dissatisfied	Satisfied	Dissatisfied	Satisfied
	Percent (%)			
**Age (in years)**				
60–69	30.70	69.30	32.51	67.49
70–79	29.52	70.48	35.79	64.21
80+	30.78	69.22	35.07	64.93
**Educational status**				
No/primary	35.24	64.76	36.66	63.34
Secondary	24.79	75.21	16.68	83.32
Higher	18.43	81.57	15.27	84.73
**Working status**				
Never	32.98	67.02	31.93	68.07
Not	34.04	65.96	36.17	63.83
Yes	30.82	69.18	35.62	64.38
Retired	17.22	82.78	18.29	81.71
**Marital status**				
Currently married	29.76	70.24	29.58	70.42
Currently unmarried^#^	32.87	67.13	37.10	62.90
**Living arrangement**				
Living alone	45.68	54.32	48.27	51.73
Living with spouse	31.99	68.01	31.07	68.93
Living with others^##^	29.27	70.73	32.66	67.34
**Social participation**				
No	33.72	66.28	38.05	61.95
Yes	27.82	72.18	29.62	70.38
**Physical activity**				
No	29.90	70.10	33.53	66.47
Yes	31.00	69.00	34.42	65.58
**SRH**				
Good	26.92	73.08	30.26	69.74
Poor	42.33	57.67	43.70	56.30
**Multimorbid**				
No	29.79	70.21	34.99	65.01
Yes	32.34	67.66	30.05	69.95
**Tobacco use**				
No	27.29	72.71	32.25	67.75
Yes	32.38	67.62	38.97	61.03
**Alcohol consumption**				
No	29.12	70.88	33.59	66.41
Yes	33.47	66.53	39.56	60.44
**ADL difficulty**				
No	29.40	70.6	32.02	67.98
Yes	34.19	65.81	38.89	61.11
**IADL difficulty**				
No	28.24	71.76	30.45	69.55
Yes	33.85	66.15	36.37	63.63
**MPCE quintile**				
Poorest	35.79	64.21	40.49	59.51
Poorer	31.21	68.79	36.63	63.37
Middle	28.78	71.22	31.59	68.41
Richer	27.20	72.80	29.42	70.58
Richest	28.07	71.93	28.26	71.74
**Religion**				
Hindu	30.58	69.42	33.66	66.34
Muslim	29.20	70.80	35.78	64.22
Others	29.28	70.72	31.69	68.31
**Caste**				
SC/ST	37.25	62.75	40.00	60.00
OBC	29.96	70.04	33.04	66.96
Others	24.30	75.70	28.75	71.25
**Place of residence**				
Urban	25.33	74.67	27.79	72.21
Rural	32.21	67.79	36.37	63.63
**Region**				
North	32.75	67.25	34.70	65.30
Central	30.90	69.10	34.55	65.45
East	32.43	67.57	40.38	59.62
Northeast	21.68	78.32	29.97	70.03
South	39.30	60.70	39.33	60.67
West	14.86	85.14	17.12	82.88
Total	30.35	69.65	33.75	66.25

% Weighted percentage, Individual sampling weights given in LASI wave 1, 2017–18 were applied.

*SRH* self-rated health, *ADL* activities of daily living, *IADL* instrumental activities of daily living, *MPCE* monthly per capita consumption expenditure.

^#^Currently unmarried category comprises widowed, divorced, separated, deserted and never married older individuals.

^##^Category of living with others consists of those older adults who were co-residing either with their spouse and children, living with spouse and other relatives, and living with children and/or other relatives.

### Multivariable regression analyses of life satisfaction among older adults

Table 3 shows the results from the multivariable linear regression analysis, which present the models explaining older adults’ life satisfaction throughout three sets. The first set demonstrates the individual effect of each predictor i.e., social participation, marital status, living arrangements, socio-demographic, economic, and health related predictors which were controlled in all specifications. Older adults who were participating in social activities [*β* = 0.39, *p* < 0.05], and those who were married [*β* = 0.68, *p* < 0.001] experienced higher life satisfaction compared to their respective counterparts. Older adults living with their spouse [*β* = 1.73, *p* < 0.001], or living with others [*β* = 2.18, *p* < 0.001] experienced higher levels of life satisfaction than older individuals living alone. No significant association was observed among the sex of the older adults and their life satisfaction. Older individuals with secondary level of education [*β* = 1.70, *p* < 0.001] and higher level of education [*β* = 1.66, *p* < 0.001] experienced higher life satisfaction compared to those older adults who were either illiterate or completed schooling up to primary level. In comparison to never worked older adults, retired individuals have slightly higher life satisfaction [*β* = 0.79, *p* < 0.05]; whereas individuals who were currently not engaged in any work activity experienced lower level of life satisfaction [*β* = − 0.54, *p* < 0.05].

**Table 3:** Association of social participation and other variables with life satisfaction among older adults, LASI Wave 1, 2017–18.

Variables	Model 1 β (95% CI)	Model 2 β (95% CI)	Model 3 β (95% CI)
**Social participation**			
No	Ref		
Yes	0.39* (0.09–0.69)		
**Marital status**			
Currently Unmarried^#^	Ref		Ref
Currently Married	0.68*** (0.29–1.06)		0.67*** (0.28–1.05)
**Living arrangement**			
Living alone	Ref	Ref	
Living with spouse	1.73*** (0.90–2.57)	1.74*** (0.90–2.57)	
Living with others^##^	2.18*** (1.42–2.94)	2.19*** (1.42–2.95)	
**Age (in years)**			
60–69	Ref	Ref	Ref
70–79	0.36* (0.00–0.73)	0.36* (0.00–0.73)	0.37* (0.01–0.73)
80+	0.98** (0.34–1.61)	0.99** (0.35–1.63)	0.99** (0.35–1.62)
**Sex**			
Male	Ref	Ref	Ref
Female	− 0.13 (− 0.50 to 0.23)	− 0.13 (− 0.50 to 0.23)	− 0.13 (− 0.50 to 0.23)
**Educational status**			
No/primary	Ref	Ref	Ref
Secondary	1.70*** (1.18–2.21)	1.69*** (1.18–2.21)	1.70*** (1.18–2.21)
Higher	1.66*** (0.99–2.32)	1.67*** (1.00–2.33)	1.66*** (1.00–2.32)
**Working status**			
Never	Ref	Ref	Ref
Not	− 0.54* (− 1.02 to − 0.06)	− 0.54* (− 1.02 to − 0.06)	− 0.54* (− 1.02 to − 0.06)
Yes	− 0.24 (− 0.74 to 0.25)	− 0.24 (− 0.74 to 0.25)	− 0.24 (− 0.74 to 0.25)
Retired	0.79* (0.09–1.50)	0.79* (0.09–1.50)	0.80* (0.09–1.50)
**Physical activity**			
No	Ref	Ref	Ref
Yes	− 0.72*** (− 1.12 to − 0.33)	− 0.72*** (− 1.12 to − 0.33)	− 0.72*** (− 1.11 to − 0.33)
**Tobacco**			
No	Ref	Ref	Ref
Yes	− 0.71*** (− 1.01 to − 0.41)	− 0.71*** (− 1.01 to − 0.41)	− 0.71*** (− 1.01 to − 0.41)
**Alcohol**			
No	Ref	Ref	Ref
Yes	− 0.20 (− 0.57 to 0.18)	− 0.20 (− 0.57 to 0.18)	− 0.20 (− 0.58 to 0.17)
**SRH**			
Good	Ref	Ref	Ref
Poor	− 2.18*** (− 2.57 to − 1.79)	− 2.17*** (− 2.56 to − 1.79)	− 2.17*** (− 2.56 to − 1.78)
**Multimorbid**			
No	Ref	Ref	Ref
Yes	0.08 (− 0.33 to 0.48)	0.07 (− 0.33 to 0.48)	0.07 (− 0.33 to 0.48)
**ADL difficulty**			
No	Ref	Ref	Ref
Yes	− 0.99*** (− 1.39 to − 0.59)	− 0.99*** (− 1.39 to − 0.59)	− 0.99*** (− 1.40 to − 0.59)
**IADL difficulty**			
No	Ref	Ref	Ref
Yes	− 0.39* (− 0.76 to − 0.02)	− 0.39* (− 0.77 to − 0.02)	− 0.39* (− 0.76 to − 0.02)
**MPCE quintile**			
Poorest	Ref	Ref	Ref
Poorer	0.49* (0.06–0.92)	0.49* (0.06–0.92)	0.49* (0.06–0.92)
Middle	1.05*** (0.60–1.50)	1.05*** (0.60–1.50)	1.05*** (0.60–1.50)
Richer	1.37*** (0.83–1.91)	1.37*** (0.83–1.90)	1.37*** (0.84–1.91)
Richest	1.19*** (0.64–1.73)	1.19*** (0.64–1.73)	1.19*** (0.65–1.73)
**Religion**			
Hindu	Ref	Ref	Ref
Muslim	− 0.14 (− 0.57 to 0.28)	− 0.14 (− 0.56 to 0.28)	− 0.15 (− 0.57 to 0.28)
Others	0.24 (− 0.29 to 0.77)	0.24 (− 0.29 to 0.77)	0.25 (− 0.29 to 0.78)
**Caste**			
SC/ST	Ref	Ref	Ref
OBC	0.92*** (0.58–1.26)	0.92*** (0.58–1.27)	0.92*** (0.58–1.26)
Others	0.90*** (0.51–1.29)	0.90*** (0.51–1.29)	0.90*** (0.51–1.29)
**Place of residence**			
Urban	Ref	Ref	Ref
Rural	− 0.27 (− 0.66 to 0.13)	− 0.26 (− 0.65 to 0.13)	− 0.26 (− 0.66 to 0.13)
**Region**			
North	Ref	Ref	Ref
Central	0.41 (− 0.00 to 0.83)	0.42 (− 0.00 to 0.83)	0.42* (0.00–0.83)
East	0.23 (− 0.13 to 0.60)	0.23 (− 0.13 to 0.60)	0.24 (− 0.13 to 0.60)
Northeast	0.78*** (0.37–1.20)	0.78*** (0.37–1.20)	0.78*** (0.36–1.19)
West	− 1.19*** (− 1.69 to − 0.69)	− 1.19*** (− 1.69 to − 0.69)	− 1.19*** (− 1.70 to − 0.69)
South	3.75*** (3.33–4.18)	3.75*** (3.33–4.18)	3.75*** (3.33–4.18)
**Social participation *X* Marital status**			
No/ Unmarried		Ref	
No/ Married		0.79*** (0.34–1.25)	
Yes/ Unmarried^#^		0.53 (− 0.05 to 1.11)	
Yes/ Married		1.10*** (0.67–1.54)	
**Social participation *X* Living arrangement**			
No/Living alone			Ref
No/Living with spouse			1.69** (0.58–2.81)
No/Living with others^##^			1.94*** (0.91–2.96)
Yes/Living alone			0.02 (− 1.33 to 1.36)
Yes/Living with spouse			1.81** (0.68–2.94)
Yes/Living with others^##^			2.43*** (1.37–3.50)

Ref.: Reference Category; *if *p* < 0.05, **if *p* < 0.01, ***if *p* < 0.001.

*CI* confidence interval, *X* interaction, *SRH* self-rated health, *ADL* activities of daily living, *IADL* instrumental activities of daily living, *MPCE* monthly per capita consumption expenditure.

^#^Currently unmarried category comprises widowed, divorced, separated, deserted and never married older individuals.

^##^Category of living with others consists of those older adults who were co-residing either with their spouse and children, living with spouse and other relatives, and living with children and/or other relatives.

Notably, those engaged in physical activities [*β* = − 0.72, *p* < 0.001] experienced lower life satisfaction. Throughout all the models, those older adults who were consuming tobacco experienced lower life satisfaction [*β* = − 0.71, *p* < 0.001]. Older adults with poor self-rated health [*β* = − 2.17, *p* < 0.001]; having difficulty in performing ADL [*β* = − 0.99, *p* < 0.001]; and IADL [*β* = − 0.39, *p* < 0.05] experienced lower level of life satisfaction. Multimorbidity conditions of older adults did not play a significant role in life satisfaction. Older adults who belonged to higher MPCE quintiles experienced higher level of life satisfaction [*β* = 0.49, p < 0.05; *β* = 1.05, *p* < 0.001; *β* = 1.37, *p* < 0.001; and *β* = 1.19, *p* < 0.001 for poorer, middle, richer, and richest MPCE quintiles respectively] in comparison to older individuals in the poorest quintile. Individuals belonging to the OBC and other castes experienced higher life satisfaction.

The interaction effect of social participation and marital status showed that married older adults, irrespective of their social participation were having higher life satisfaction [married and not participated in social activities: *β* = 0.79, *p* < 0.001; married and participated in social activities: *β* = 1.10, *p* < 0.001], compared to unmarried older adults who did not participate in any social activities. Similarly, interaction of social participation and living arrangements showed significant association with life satisfaction. With reference to older adults who were living alone and not involved in social participation, those older individuals who were either living with their spouse [*β* = 1.69, *p* < 0.01]; or living with others [*β* = 1.94, *p* < 0.001] and not involved in social participation, and older individuals who were involved in social participation and living with their spouse [*β* = 1.81, *p* < 0.01], or living with others [*β* = 2.43, *p* < 0.001] were associated with significantly higher level of life satisfaction.

## Discussion

This study examined the role played by social participation, marital status, and living arrangements on life satisfaction among older Indian adults. It is documented that the life satisfaction of people in Asia varies from country to country. Ngoo and colleagues (2015) analyzed life satisfaction across 28 Asian countries. They noted that the people of Maldives in South Asia have the highest life satisfaction (mean of 64.4), followed by Indonesia (64.3), and the Philippines (63.6) in Southeast Asia, Bhutan (62.8) and Sri Lanka (62.1) in South Asia. On the other side, the people in Turkmenistan, Uzbekistan and Mongolia (in Central and West Asia), Myanmar (Southeast Asia), and China (East Asia) have the lowest life satisfaction mean ranging from 47.9 to 51.2. India was ranked ninth (mean of 60.6) for life satisfaction among the 28 countries^3^. The current study estimates that 69.6 percent of older men and 66.2 percent of older women were satisfied with their life. The regional variations and differences in the estimates of life satisfaction in the current and previous studies might have occurred due to different standards of living, governmental roles and changing values attributed to family institution within Asian countries. The different methodology and scales of life satisfaction might have also led to the observed variations.

While examining the overall sex differentials in life satisfaction, current study clearly showed higher life satisfaction among older men than women in almost all the background characteristics. However, living with a spouse or having secondary and higher education were exceptional categories where the older men have slightly less life satisfaction than their women counterparts. Thus, sex differential in life satisfaction is an arena for future research in particular Indian context. Further, social participation is usually assumed to be beneficial to older adults' life satisfaction, and some previous empirical studies have supported this theoretical proposition. However, how older adults' marital status and living arrangements moderate the relationship between social participation and life satisfaction is yet to be explored, which is addressed in the present study using India’s nationally representative data from the LASI wave 1.

Firstly, the effects of social participation on life satisfaction of Indian older adults were examined, using multivariable linear regression that adjusts for a broad range of covariates. There is evidence of a significant effect of social participation on life satisfaction, but these appeared to be weaker once controlled for a range of covariates in the regression model. Although the result was not so robust, it showed a positive association that can validate the rationality of activity theory. This finding is also supported by previous empirical studies^19,20^. Havighurst and Albrecht^15^, in their activity theory of aging, stated that staying occupied and involved in activities is necessary for having a satisfying life at later ages. Being active at later ages benefits the quality of life because social participation generates social interaction and increases social support and emotional closeness, which may help reduce tension and meet the psychological needs of an individual.

The second most important predictor of life satisfaction is older adults’ marital status. The study found higher life satisfaction among married older adults than their unmarried counterparts. This finding is supported by a recent study in which marital status turns out to be the most crucial predictor of life satisfaction among most Asian countries^47^. The interaction of social participation with marital status showed that in comparison to unmarried older adults who were not engaged in social activities, married older adults with no social participation have a higher level of life satisfaction. The literature showed that presence of a spouse had provided relatively greater life satisfaction for older individuals^48^.

The interaction of social participation with marital status also revealed that participation in social activities had boosted life satisfaction to a greater level among married older adults. At the same time, unmarried older adults with an engagement in social participation were found to be an insignificant predictor of life satisfaction in this analysis. So, being married is strongly related to better life satisfaction among older Indian adults. Marital transition, particularly divorce or widowhood, disrupts family relationships, and a person loses some social roles, exhibiting lower engagement in social participation. Such social disengagement brings loneliness and loss of satisfaction with life. Khodabakhsh^4^ stated that in Asia, a failed marriage has adverse consequences on life, negatively impacting life satisfaction among older adults.

Researchers found that late-life widowhood alters the level of social participation^49^. However, findings from previous empirical studies on social participation during widowhood were inconsistent. Widowed older adults reduced their participation in social activities^50,51^ or reported less social engagement^49,52^ after the death of a spouse. Nonetheless, some studies showed that social engagement among widowed older adults became increasingly salient after spousal death, thereby increased bereaved persons’ level of social involvement. Utz and colleague^53^ found that levels of social participation among widowed older adults were higher than non-widowed older adults. They also pointed out that increased support from friends and relatives in the realm of social participation helps older adults cope with spousal loss. In short, life satisfaction may be beneficially affected by social participation, especially among unmarried older individuals.

Consistent with the general expectation, the study findings confirmed that older adults who lived with a spouse or lived with other family members (including spouses or children) had higher life satisfaction than those who lived alone. This finding is coherent with several empirical studies conducted in Asian countries that confirmed the relationship between types of living arrangements and life satisfaction in older adults^24,33,54–58^ Kim et al.^24^, Shin and Sok^55^, and Roh and Weaon^58^ reported significant association between living alone and lower life satisfaction among older Korean population. Similarly, study evidence based on Malaysian and Chinese older population database also confirmed that co-residency with family members was associated with better life satisfaction compared to living alone^33,59^. Kamiya and Herto^60^ noted that in most underdeveloped and developing countries, older persons' coresidential living arrangement is an essential element of the flow of financial, emotional, and care support between family members, which affects the well-being of older individuals. Such emotional support and care are often received through social participation.

Furthermore, confirming our final hypothesis, the results highlighted that older adults who were not involved in social participation but resided with a spouse or any other family members (including spouses or children) were significantly associated with better life satisfaction. Again, older adults who resided in similar living arrangements and participated in social activities enjoyed higher levels of life satisfaction. Hence, our study strongly supported the significant role of living arrangements in improving aged people's life satisfaction along with the interaction effect of social participation. Overall, it is observed that social participation coupled with family care and support is one way through which older adults can potentially enhance their life satisfaction.

### Policy, practice and research implications

Our findings emphasize the importance of family and social participation for older people's life satisfaction. Across India, several non-profit organizations (NGOs), Viz., HelpAge India, VridhCare, Abhoy Mission, Manavlok, Asha Kiran, etc., are actively working for the well-being and empowerment of socially disadvantaged older people (those living alone/ divorced/ widowed/ separated). With the involvement of NGOs and social workers, the government can establish community, village, or ward-level clubs that can organize productive and consumptive activities to improve the well-being of disadvantaged older people. To address the multidimensionality of active aging, we suggest four types of participation: volunteering, participation in old-age educational activities, participation in social leisure activities, and, finally, religious participation (especially for widows). All these activities must be performed through well-established clubs, which can help to develop and maintain a more comprehensive social network among older people of the same age cohort. Future studies must explore the alternative social support mechanisms for older Indian adults’ overall life satisfaction, i.e., on what platform it can be implemented and also what effective strategies can be developed for this purpose.

### Limitations of the study

In spite of its significant theoretical and methodological contributions, this study has several limitations. First, since this is a cross-sectional study, the possibility of reverse causality cannot be ruled out. For instance, lower levels of life satisfaction may lead older people to reduce participation in social activities. Hence, care must be taken while making the causal inferences about the relationship between social participation of aged people and their life satisfaction. Second, the use of categorical data to represent a great deal of richer continuous variables in the current analysis might have influenced the findings. Third, self-report nature of several explanatory variables might be subject to recall and response biases.

Fourth, there are several potentially confounding variables that are not considered in this study. For example, few researchers, have emphasized that the perceived quality of social networks^23,25^ or satisfaction with social support^9^ are more important determinants of life satisfaction than the size of social networks. In our analysis, such determinants have not been taken into account due to the shortcomings stemming from the limited information about the frequency of social participation and perceived quality of social interactions that were not available in LASI data. Availability of such information can allow to do in-depth research in future. Moreover, quality of marriages as well as diet pattern of older individuals may have significant effect on their satisfaction with life, which need to be further investigated in future research. The mechanisms underlying the observed associations and interactions should also be explored further in future studies.

Despite these limitations, this study has several strengths, including its population-based design, and the assessment of life satisfaction with an internationally validated scale and inclusion of a wide range of possibly confounding variables in the analysis.

## Conclusion

Along with social participation, the study findings assert the importance of being in a marital union and co-residential living arrangements for the overall life satisfaction of older Indians. Additionally, the study findings supported the hypothesis of activity theory. In this study, older adults exposed to vulnerabilities like marital disruptions, lower educational status, poor household consumption quintiles, poor health conditions, and living alone were at a higher risk of life dissatisfaction because social withdrawal occurs among such vulnerable groups. Establishing social clubs and initiating vivid activities, especially for those older adults exposed to such vulnerabilities, will help improve their overall life satisfaction. The information deriving from this study can be used to advocate programs and service delivery for older adults who are either currently not in a marital union or living alone and suffering from life dissatisfaction. Such program execution will help to seek or maintain meaningful quality of life and create a more positive atmosphere for older adults' overall life satisfaction.

## Data Availability

The study uses secondary data which is available on reasonable request through https://www.iipsindia.ac.in/content/lasi-wave-i.
